# ABCE1 Regulates RNase L-Induced Autophagy during Viral Infections

**DOI:** 10.3390/v13020315

**Published:** 2021-02-18

**Authors:** Barkha Ramnani, Praveen Manivannan, Sarah Jaggernauth, Krishnamurthy Malathi

**Affiliations:** Department of Biological Sciences, University of Toledo, 2801 West Bancroft Street, Toledo, OH 43606, USA; Barkha.Ramnani@rockets.utoledo.edu (B.R.); Praveen.Manivannan@rockets.utoledo.edu (P.M.); Sarah.Jaggernauth@rockets.utoledo.edu (S.J.)

**Keywords:** RNase L, ABCE1, RLI, autophagy, interferon, apoptosis

## Abstract

Host response to a viral infection includes the production of type I interferon (IFN) and the induction of interferon-stimulated genes that have broad antiviral effects. One of the key antiviral effectors is the IFN-inducible oligoadenylate synthetase/ribonuclease L (OAS/RNase L) pathway, which is activated by double-stranded RNA to synthesize unique oligoadenylates, 2-5A, to activate RNase L. RNase L exerts an antiviral effect by cleaving diverse RNA substrates, limiting viral replication; many viruses have evolved mechanisms to counteract the OAS/RNase L pathway. Here, we show that the ATP-binding cassette E1 (ABCE1) transporter, identified as an inhibitor of RNase L, regulates RNase L activity and RNase L-induced autophagy during viral infections. ABCE1 knockdown cells show increased RNase L activity when activated by 2-5A. Compared to parental cells, the autophagy-inducing activity of RNase L in ABCE1-depleted cells is enhanced with early onset. RNase L activation in ABCE1-depleted cells inhibits cellular proliferation and sensitizes cells to apoptosis. Increased activity of caspase-3 causes premature cleavage of autophagy protein, Beclin-1, promoting a switch from autophagy to apoptosis. ABCE1 regulates autophagy during EMCV infection, and enhanced autophagy in ABCE1 knockdown cells promotes EMCV replication. We identify ABCE1 as a host protein that inhibits the OAS/RNase L pathway by regulating RNase L activity, potentially affecting antiviral effects.

## 1. Introduction

Degrading viral and cellular RNAs required for viral replication is an evolutionarily conserved antiviral mechanism. In higher vertebrates, this process is regulated by interferon (IFN), produced during a viral infection through the activation of the ubiquitous cellular latent endoribonuclease, ribonuclease L (RNase L). The 2′,5′-oligoadenylate synthetase (OAS)/RNase L system is an innate immune pathway that responds to the double-stranded RNAs (dsRNAs) that serve as pathogen-associated molecular patterns (PAMPs) to induce the degradation of viral and cellular RNAs, thereby blocking the viral infection [1,2,3]. Type I IFN, produced and secreted by a virus-infected cell signal through the type I IFN receptor, activates JAK-STAT signaling and induces the expression of interferon-stimulated genes (ISGs), including oligoadenylate synthetases (OAS), that together establish the antiviral state [4,5]. OAS1–3 isoforms are expressed at varying levels in different cell types, and on activation by dsRNA PAMPs, certain OAS proteins produce 2-5A from cellular ATP [6,7,8]. 2-5A is a unique ligand that binds monomeric and latent RNase L with high affinity, causing RNase L dimerization and activation. Active RNase L cleaves diverse single-stranded RNA substrates, including viral genomes and cellular RNAs, directly impacting protein synthesis and limiting viral replication [3]. Activation of RNase L, through the generation of dsRNA cleavage products, amplifies IFN production, activates inflammasome, leads to autophagy, and promotes a switch from autophagy to apoptosis, affecting viral replication in cells [9,10,11,12,13].

Autophagy may function as an antiviral mechanism by activating signaling pathways to promote the elimination of viruses, or it may provide a platform to enhance viral replication [14]. Accumulation of dsRNA PAMPs during a viral infection and the expression of antiviral proteins induce apoptosis in virus-infected cells. Both autophagy and apoptosis share regulatory molecules, and cross-talk between these two pathways dictates the outcome of the viral infection [15,16]. Our previous studies show that the activation of RNase L by 2-5A induces autophagy and the small dsRNAs generated by RNase L enzyme activity promote a switch from autophagy to apoptosis by the caspase-mediated cleavage of the key autophagy protein, Beclin-1. The cleavage of Beclin-1 results in the termination of autophagy, and the cleaved Beclin-1 fragment translocates to the mitochondria and induces apoptosis [12,13].

The OAS/RNase L pathway exerts an antiviral effect against a wide range of viruses. Consequently, viruses have evolved mechanisms to counteract the antiviral activity of the OAS/RNase L pathway by antagonizing or inhibiting the activation of RNase L [17,18]. The evasion strategies target all steps of the OAS/RNase L pathway. The influenza A virus NS1 protein, vaccinia virus E3L, and the herpes simplex virus type 1 US11 protein sequester dsRNA, thereby preventing the activation of OAS and 2-5A synthesis [19,20,21]. DNA viruses like vaccinia virus, herpes simplex virus, and SV40 produce inactive or inhibitory 2-5A-like molecules that are unable to activate RNase L [22,23,24]. Theiler’s murine encephalomyelitis virus (TMEV) encodes an L* protein that directly binds to the ankyrin repeat domain of RNase L, inhibiting the binding of 2-5A to RNase L [25,26]. A highly structured region of the poliovirus genomic RNA acts as a cleavage-resistant substrate for RNase L and competitively inhibits RNase L [27]. Murine hepatitis virus (MHV) ns2, Middle East respiratory syndrome coronavirus (MERS-CoV) NS4b, and rotaviruses (RV) VP3 encode for phosphodiesterases, which degrade 2-5A and thus prevent RNase L activation [28,29,30,31]. Host cells also express phosphodiesterases PDE12, ENPP1, and AKAP7, which can degrade and regulate the turnover of 2-5A in cells [32,33,34,35,36,37]. In addition, ATP-binding cassette E1 (ABCE1), also known as RNase L inhibitor (RLI), was identified as an RNase L-interacting protein that modulates RNase L activity by preventing 2-5A binding [38].

ABCE1 (RNase L inhibitor (RLI)) is a member of the superfamily of ABC transporters but lacks a membrane-spanning domain necessary for transporter function. RLI was initially identified as a negative regulator of RNase L by directly binding to RNase L and inhibiting the binding of 2-5A [38,39]. In contrast, a recent study showed that the interaction of ABCE1 with RNase L accelerates dimerization and acts as a positive regulator of exogenous RNA decay [40]. ABCE1 is induced by EMCV and HIV and may serve as a mechanism to evade the antiviral effects of RNase L [41,42]. In addition, ABCE1 interacts with the HIV gag protein to mediate capsid assembly [43,44]. A genome-wide siRNA screen identified ABCE1 as an essential host factor that is required for the efficient translation of measles and mumps viral proteins. In that context, ABCE1 knockdown only inhibited the translation of measles virus mRNAs and not that of cellular RNAs, demonstrating an important role for ABCE1 in measles virus pathogenesis [45]. ABCE1 is conserved evolutionarily from lower eukaryotes, while RNase L function is restricted to higher vertebrates, suggesting additional cellular roles of ABCE1. Accordingly, studies have shown the role of ABCE1 in the regulation of translation, ribosome recycling, and homeostasis by interacting with translation initiation and release factors and ribosomal subunits [46,47,48,49,50,51]. ABCE1 dissociates ribosomes during translation termination and mRNA surveillance on stalled ribosomes [49,52]. Knockdown of ABCE1 inhibits proliferation and migration and induces apoptosis in small cell lung cancer, breast cancer, and esophageal cancer cells [53,54,55]. Here, we show that ABCE1 inhibits RNase L activity; cells with reduced ABCE1 levels transfected with 2-5A show increased RNase L activity that corresponds with early onset of autophagy. Enhanced RNase L activity in ABCE1 knockdown cells inhibits proliferation and induces apoptosis by the caspase-3-mediated cleavage of Beclin-1, terminating autophagy. Furthermore, ABCE1 promotes EMCV pathogenesis by regulating autophagy. These studies identify a novel role of ABCE1 in regulating RNase L-induced autophagy during viral infections.

## 2. Materials and Methods

### 2.1. Chemicals, Reagents, and Antibodies

The chemicals, unless indicated otherwise, were from Sigma-Aldrich (St. Louis, MO, USA). The antibodies to LC3, SQSTM1/p62, cleaved PARP, cleaved caspase-3, β-actin, and Beclin-1 were purchased from Cell Signaling Technology (Danvers, MA, USA); the ABCE1 (RLI) antibody was purchased from Abcam (Cambridge, MA, USA), and 3D Pol was purchased from Santa Cruz Biotechnology (Santa Cruz, Dallas, TX, USA). Anti-mouse IgG and anti-rabbit IgG HRP-linked secondary antibodies were from Cell Signaling. The ECL reagents were from Boston Bioproducts (Ashland, MA, USA) and Bio-Rad Labs (Hercules, CA, USA). Puromycin was purchased from Thermo Fisher Scientific (Waltham, MA, USA) and used as described. Bafilomycin A1 was from Enzo Life Sciences (Farmingdale, NY, USA), and 3-methyladenine was from Sigma-Aldrich (St. Louis, MO, USA). The GFP-LC3 plasmid was provided by Isei Tanida (via Addgene, Watertown, MA, USA) [56]. The encephalomyocarditis virus (EMCV K strain) and the monoclonal antibody to human RNase L were a kind gift from Robert Silverman (Cleveland Clinic, Cleveland, OH, USA). Poly I:C was purchased from Calbiochem (San Diego, CA, USA). 2-5A (p_3_(A2′p)*_n_*A, where *n* = 1 to >3) was prepared enzymatically from ATP and recombinant 2-5A synthetase (a generous gift from Rune Hartmann, University of Aarhus, Aarhus, Denmark) as described previously [57]. Briefly, poly I:C-agarose was bound with recombinant OAS1 and incubated with a buffer containing ATP for 20 h at 37 °C. The reaction mix was clarified by centrifugation and passed through a 3 kDa centriprep filter (Millipore Sigma, MO, USA), and the 2-5A preparation obtained was applied to cells complexed with Lipofectamine 2000 (Invitrogen, Carlsbad, CA, USA) [58].

### 2.2. Cell Culture and Transfections

The human fibrosarcoma cell line HT1080 (a gift from Ganes Sen, Cleveland Clinic, Cleveland, OH, USA), RNase L KO, ABCE1 KD, ABCE1 KD/RNase L KO, and the mouse fibroblast cell line L929 (a gift from Douglas Leaman, Wright State University) were cultured in Dulbecco’s modified minimal essential medium with 10% fetal bovine serum (Sigma-Aldrich, St. Louis, MO, USA), 100 μg/mL penicillin/streptomycin, 2 mM l-glutamine, and nonessential amino acids. RNase L KO cells were generated with CRISPR/Cas9 gene editing as described previously [59]. The cells were maintained in 95% air, 5% CO_2_ at 37 °C. Transfection of 2-5A (10 µM) or poly I:C (2 µg/mL) was performed using Lipofectamine 2000 (Invitrogen, Carlsbad, CA, USA) according to the manufacturer’s protocol. Briefly, the cells were plated one day before transfection so that the cells were 80–90% confluent at the time of transfection. Poly I:C or 2-5A was diluted into a serum-free medium and then mixed with Lipofectamine 2000 reagent for 20 min before being added to cells in growth media and incubated for indicated times.

### 2.3. Generation of ABCE1 Knockdown Cells

ABCE1 (RLI) was knocked down in HT1080 WT by transfecting pSUPER plasmid with short hairpin RNA homologous to ABCE1 (RLI) along with pBabe-puro as previously described [60]. Individual clones were generated by limiting dilution in a puromycin (1 µg/mL) containing medium, and gene knockdown was verified by immunoblot analysis using ABCE1 antibodies and normalized to β-actin levels for comparison. Two independent clones (clone 1 and clone 2) were used to determine RNase L activity, induction of autophagy, and cell viability assays to rule out clone-specific artifacts. The data obtained with clone 2 compared to clone 1 are shown in Figure S1. ABCE1 knockdown cells from early passages were used in this study as later passages showed reduced proliferation and viability, as observed in other studies [45]. To generate ABCE1 KD/RNase L KO cells, RNase L KO CRISPR/Cas9 gene edited cells were co-transfected with ABCE1 short hairpin RNA (shRNA) and pEGFP plasmid. GFP-positive individual clones obtained by limiting dilution were verified by immunoblot analysis as described above and used.

### 2.4. Measuring RNase L Activity in Intact Cells

Cells were transfected with poly I:C (2 µg/mL) or 2-5A (10 μM) using Lipofectamine 2000 reagent and after 6 h, total RNA was isolated using Trizol reagent (Invitrogen, Thermo Fisher Scientific, Waltham, MA, USA). Total RNA was resolved on RNA chips and analyzed with Bioanalyzer 2100 (Agilent Technologies, Santa Clara, CA, USA) as described previously [12]. RNase L activity was measured as the cleavage of total RNA by estimating the integrity by the RNA integrity number (RIN) [61].

### 2.5. Quantification of Autophagy

Cells were transfected with GFP-LC3 and 24 h later with 2-5A (1 0µM) using Lipofectamine 2000 and imaged at indicated times using an Olympus IX81 inverted fluorescence microscope. Cytoplasmic GFP-LC3 puncta (>10 puncta/cell) were considered autophagic and manually counted for at least 100 randomly selected cells per preparation from three independent experiments. Autophagy was quantified as a percent of GFP-LC3-positive cells showing puncta (>10 puncta/cell). The kinetics of the induction of autophagy in live cells was determined using the CYTO-ID autophagy detection kit (Enzo Life Sciences, Farmingdale, NY, USA) under a confocal microscope. Briefly, cells (1 × 10^5^) were seeded on coverslips in a 12-well plate and transfected with 2-5A (10 µM) using Lipofectamine 2000 reagent. The Cyto-ID Green Autophagy Detection Reagent, which is excitable at 488 nm in autophagic vacuoles produced during autophagy, was added and imaged at indicated times. The percent of cells showing autophagic vacuoles from three random fields was counted. A minimum of 100 cells per preparation were quantified in three independent experiments.

### 2.6. Cell Viability and Caspase 3/7 Assays

The viability of cells was determined using the colorimetric CellTiter 96 Aqueous Cell Proliferation Assay (Promega, Madison, WI, USA). Briefly, cells (8 × 10^3^) were seeded into 96-well plates and transfected with 2-5A (10 µM). At indicated times, 20 µL of tetrazolium salt (MTS reagent) was added and incubated at 37 °C. Absorbance was measured at 490 nm with a 96-well plate reader (model Spectra Max iD5; Molecular Devices, Menlo Park, CA, USA). Cell viability was normalized against mock-treated cells. The percent of viable cells was determined by staining in a 0.4% trypan blue solution (Life Technologies, Carlsbad, CA, USA) and estimating viable cells that exclude dye uptake using a hemocytometer and normalized to mock-treated cells. For caspase 3/7 assay, cells were grown in black-walled 96-well plates with transparent bottoms (Costar) and transfected with 2-5A (10 µM) using Lipofectamine 2000. At indicated times, caspase3/7 activity in lysates was measured using the ApoONE homogenous caspase-3 and -7 assay kit (Promega, Madison, WI, USA) and normalized to control mock-treated cells. Experiments were performed in triplicate and shown as mean ± SD.

### 2.7. Cell Death Assays

Cell death was analyzed in real time using dual dyes and an IncuCyte S3 Live-Cell imaging system (Essen BioScience, Ann Arbor, MI, USA). Cells (8 × 10^3^) were seeded into a 96-well plate and transfected with 2-5A (10 µM) using Lipofectamine 2000 reagent. Cells were incubated with 250 nM Sytox-Green cell-impermeable nucleic acid dye (ThermoFisher Scientific, Waltham, MA, USA) that indicates dead cells and 250 nM of SytoTM 60-Red cell-permeable dye (ThermoFisher scientific, Waltham, MA, USA), which quantifies the total number of cells present in each field, and images were obtained in real time as indicated. Cell death was quantified as the percent of Sytox-Green-positive dead cells normalized to the total number of cells that were stained Sytox-Red positive at each time point using IncuCyte S3 software. Results shown are representative of four values per well performed in triplicate from three experimental samples and shown as mean ± SD.

### 2.8. Immunoblotting

Cells were washed in ice-cold PBS and lysed in an NP-40 lysis buffer containing 0.5% NP-40, 90 mM KCl, 5 mM magnesium acetate, 20 mM Tris (pH 7.5), 5 mM β-mercaptoethanol, 0.1 M phenylmethylsulfonyl fluoride (PMSF), 0.2 mM sodium orthovanadate, 50 mM NaF, 10 mM glycerophosphate, and a protease inhibitor (Roche Diagnostics). The lysates were clarified by centrifugation at 10,000× *g* (4 °C for 20 min). Proteins (15–60 μg per lane) were separated in polyacrylamide gels containing SDS and transferred to a nitrocellulose membrane (Bio-Rad, Hercules, CA, USA) and probed with different primary antibodies according to the manufacturer’s protocols. Membranes were washed and incubated with goat anti-mouse or goat anti-rabbit antibody tagged with horseradish peroxidase (Cell Signaling, Danvers, MA, USA) for 2 h. Immunoreactive bands were visualized using enhanced chemiluminescence reagents (Boston BioProducts, Ashland, MA, USA; Bio-Rad, Hercules, CA, USA). For determining the ratios of LC3-II/β-actin, P62/β-actin, and 3D Pol/β-actin, the intensity of each band was determined using Image J software (National Institutes of Health, Bethesda, MD, USA) and their relative intensities were calculated by normalizing to β-actin. Images were processed using Adobe Photoshop CS4 (Adobe, San Jose, CA, USA). In some instances, nonspecific lanes were cropped to generate the images and the boundaries are indicated in representative figures.

### 2.9. Virus Infections and Plaque Assays

The cells were plated into 6-well plates, and the next day, the cells were washed twice in PBS and infected with EMCV (strain k) at MOI = 1.0. After 1 h, the virus was removed and the cells were washed with PBS and replaced with a complete growth medium. In experiments with inhibitors, the cells were pretreated with 3-methyladenine (5 mM) or bafilomycin A1 (100 nM) for 1 h and infected with EMCV, and then fresh complete media with 10% FBS was added. At indicated times post infection, EMCV containing supernatants and cell pellets were harvested for virus titration. Serial dilution of supernatants or clarified cell lysates containing intracellular virus were added to the confluent monolayer of L929 cells in 12-well plates. The plates were incubated for 1 h at 37 °C. The cells were washed with PBS and overlaid with DMEM containing 0.5% carboxymethylcellulose and incubated for 24 h. The cells were fixed with 10% formaldehyde, and plaques were stained using 0.1% crystal violet and counted. The assays were performed in triplicate, and the fold change in virus titers was determined from three independent experiments and shown as mean ± SD. The expression of the viral antigen was determined on Western blots using the 3D Pol antibody (Santa Cruz Biotechnology, Dallas, TX, USA).

### 2.10. Statistical Analysis

All values are presented as mean ± SD and are representative of at least three independent experiments. Two-way ANOVA (or one-way ANOVA for a single time point) was used for determining statistical significance between groups, using Prism 8 (GraphPad) software, and *p* values of <0.05 were considered significant.

## 3. Results

### 3.1. ABCE1 (RLI) Regulates RNase L Enzyme Activity

**Figure 1:**
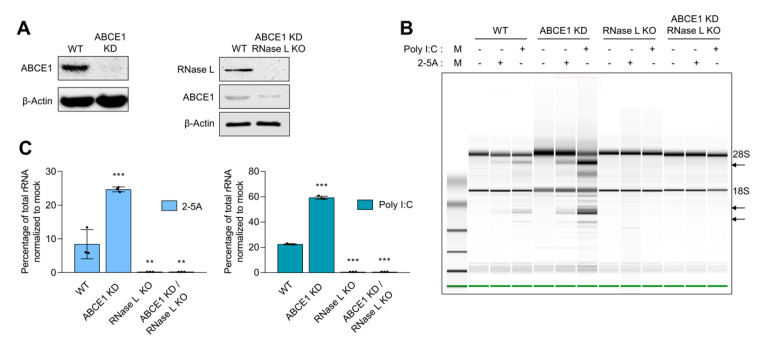
Increased ribonuclease L (RNase L) activity in cells with reduced ATP-binding cassette E1 (ABCE1) protein levels. (**A**) Knockdown of ABCE1 levels using short hairpin RNA (shRNA) in HT1080 WT cells or CRISPR/Cas9 RNase L KO cells was verified in cell lysates by immunoblotting using specific antibodies. (**B**) WT, ABCE1 KD, RNase L KO, and ABCE1 KD/RNase L KO cells were transfected with 2-5A (10 µM) or 2 µg/mL of poly I:C for 6 h, and the RNase L-mediated cleavage of rRNA (arrows) was analyzed on RNA chips using Agilent Bioanalyzer 2100. (**C**) The cleavage of rRNA was quantitated and normalized to mock-treated samples. Data are representative of at least three independent experiments and expressed as means ± SD; WT: wild-type; ** *p* < 0.01; *** *p* < 0.001.

ABCE1, also known as RLI, was identified as an inhibitor of IFN-regulated endoribonuclease, RNase L. To determine if ABCE1 regulates RNase L enzyme activity, ABCE1 knockdown stable cell lines were generated in HT1080 cells by transfecting shRNA-targeting ABCE1 and stable clones with greater than 90% reduced expression were screened and used in this study (Figure 1A). Two independent clones were evaluated for effect on RNase L enzyme activity (Figure 1 and Figure S1A). As reported in other studies, prolonged ABCE1 knockdown caused reduced cell proliferation and early passage cells were expanded and used [45]. The effect of ABCE1 on RNase L enzyme activity was determined by transfecting 2-5A, a unique ligand and activator of RNase L, and rRNA cleavage characteristic of RNase L activity was quantitated and analyzed on RNA chips. Control cells produced 8.4% rRNA cleavage, while ABCE1 knockdown (ABCE1 KD) had 3-fold increased rRNA cleavage (25%). Transfecting cells with synthetic dsRNA (poly I:C), which binds oligoadenylate synthetase (OAS) to produce 2-5A from cellular ATP, produced 22.4% rRNA cleavage in WT cells compared to 59% in ABCE1 KD cells (Figure 1B,C). As expected, RNase L KO cells generated by CRISPR/Cas9 technology showed no cleavage of rRNA with 2-5A or poly I:C transfection [58,59]. To further test that the impact of ABCE1 on RNase L activity was mediated by RNase L, we knocked down ABCE1 in RNase L KO cells using shRNA and monitored rRNA cleavage by 2-5A or poly I:C transfection (Figure 1A–C). No rRNA cleavage was observed in the ABCE1 KD/RNase L KO cells, indicating that the effect of ABCE1 was mediated by regulating RNase L activity (Figure 1B,C). These results suggest that ABCE1 regulates RNase L enzyme activity and cells with reduced levels of ABCE1 show enhanced RNase L activity in the presence of the 2-5A ligand.

### 3.2. ABCE1 Modulates RNase L-Induced Autophagy

**Figure 2:**
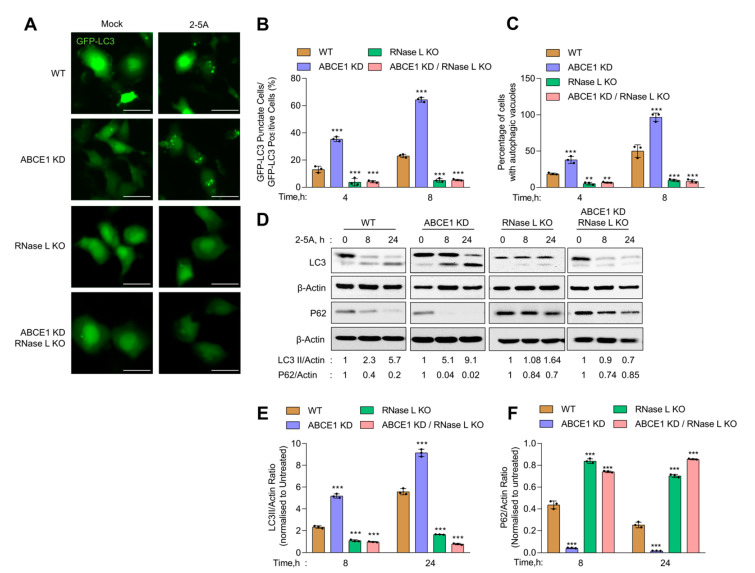
Early onset of autophagy in ABCE1 KD cells on RNase L activation. HT1080 WT, ABCE1 KD, RNase L KO, and ABCE1 KD/RNase L KO cells expressing GFP-LC3 were mock-transfected or transfected with 2-5A (10 µM) and (**A**) the formation of GFP-LC3 puncta was microscopically imaged after 8 h; scale bar 50 µm. (**B**) The percent of GFP+ cells showing puncta formation compared with mock-treated cells was analyzed. (**C**) Autophagy induction in live cells was evaluated by staining autophagic vacuoles and quantified. Results shown represent mean ± SD for the experiment performed in triplicate and shown as the percent cells showing autophagic vacuoles from randomly selected fields. (**D**) Cell lysates were harvested at indicated times, and the conversion of unconjugated LC3-I to lipidated LC3-II and degradation of p62 were monitored on immunoblots and normalized to β-actin levels. (**E**,**F**) The band intensity was calculated using Image J software, and the ratio of LC3-II/β-actin or p62/β-actin was determined and the levels were compared to WT cells. Results are representative of three independent experiments. WT: wild-type; ** *p* < 0.01; *** *p* < 0.001.

Previous work from our and other groups have shown that RNase L activation induces autophagy [11,12]. We used several assays to determine the impact of ABCE1 on RNase L-induced autophagy [62]. First, WT, ABCE1 KD, RNase L KO, and ABCE1 KD/RNase L KO cells expressing GFP-LC3 were transfected with 2-5A and the formation of distinct GFP-LC3 puncta that correspond to autophagosomes and induction of autophagy was quantified at indicated times (Figure 2A). We observed a significant increase in GFP-LC3 puncta (35.3% of GFP+ cells) as early as 4 h post transfection in ABCE1 KD cells compared to WT cells (10.4%). A similar increase in GFP-LC3 puncta was observed in another ABCE1 KD clone 2 (Figure S1B). In comparison, GFP-LC3 puncta were observed in only 3.7% of RNase L KO and 5.1% of ABCE1 KD/RNase L KO cells at the same time point. After 8 h, the number of GFP-LC3 puncta increased to 64.3% in ABCE1 KD cells and 18.9% in WT cells. However, the numbers remained largely unchanged in RNase L KO and ABCE1 KD/RNase L KO cells (Figure 2B). We then measured the accumulation of autophagic vacuoles in live cells transfected with 2-5A by staining with a fluorescent dye that selectively labels autophagic vacuoles. Compared to WT cells, ABCE1 KD cells showed a 2-fold increase in autophagic vacuole accumulation 4 h and 8 h post 2-5A treatment, while RNase L KO and ABCE1 KD/RNase L KO cells showed very low levels (Figure 2C). During autophagy, LC3-I is cleaved and lipidated to LC3-II, which is associated with autophagosomes. We found that ABCE1 KD enhanced autophagy in 2-5A-transfected cells, as shown by increased levels of LC3-II on immunoblots as early as 8 h compared to WT cells (Figure 2D). As shown previously, cells lacking RNase L did not induce autophagy with 2-5A treatment [12,13], and cells lacking both ABCE1 and RNase L did not induce autophagy by 2-5A transfection. Consistent with enhanced and early onset of autophagy, 2-5A treatment of ABCE1 KD cells caused pronounced degradation of p62 (SQSTM1), an LC3-binding protein that is degraded on autophagosome-lysosome fusion, compared to WT, RNase L KO, or ABCE1 KD/RNase L KO cells (Figure 2D–F). Taken together, these results suggest that the knockdown of ABCE1 induced early autophagy in RNase L-activated cells and these effects of ABCE1 are mediated by a direct effect on RNase L.

### 3.3. RNase L Activation Sensitizes ABCE1 Knockdown Cells to Apoptosis

**Figure 3:**
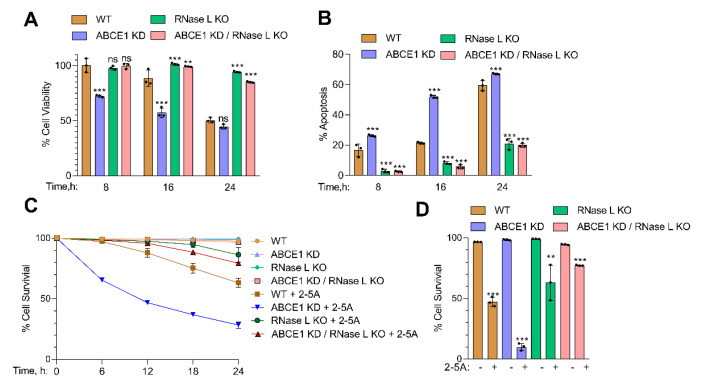
Involvement of ABCE1 in RNase L-induced apoptosis in response to 2-5A treatment. HT1080 WT, ABCE1 KD, RNase L KO, and ABCE1 KD/RNase L KO cells were transfected with 10 µM of 2-5A for indicated times and (**A**) cell viability was measured using MTS reagent. (**B**) The percent of apoptotic cells was determined by trypan blue dye exclusion assay. Results are representative of three independent experiments performed in triplicate and shown as mean ± SD and compared to WT cells; and (**C**) real-time cell viability was measured over time using a dual dye monitoring system in cells transfected with 2-5A and compared to mock-transfected cells. The percent cell survival in each well was determined by quantitating dead cells and normalized to the total number of cells at each time point. Data are representative of four values per well performed in triplicate from three experimental samples and shown as mean ± SD. (**D**) Quantitation of percent cell survival at 30 h after 2-5A transfection normalized to mock-transfected cells. WT: wild-type; ** *p* < 0.01; *** *p* < 0.001; ns: not significant.

Activation of RNase L by 2-5A induces cell death by apoptosis, and the byproducts of RNase L enzyme activity promote a switch from autophagy to apoptosis [13,63]. To determine if the increased RNase L enzyme activity in ABCE1 KD cells affected cell viability, WT, ABCE1 KD, RNase L KO, and ABCE1 KD/RNase L KO cells were transfected with 2-5A. At indicated times, cell viability was determined by MTS assay and trypan blue exclusion assays. After 8 h, ABCE1 KD cells showed 72% cell viability compared to minimal loss of viability in WT, RNase L KO, and ABCE1 KD/RNase L KO cells. The viability of ABCE1 KD cells was further reduced at 16 h to 57%, compared to 88% in WT cells, and the loss of viability continued to decrease at 24 h to 44% in ABCE1 KD cells while 62% of the WT cells were viable. Similarly, ABCE1 KD clone 2 showed 48% cell viability at 24 h (Figure S1C). No significant loss of viability was observed in RNase L KO and ABCE1 KD/RNase L KO cells (Figure 3A).

ABCE1 KD cells showed increased cell death from 26% to 67% in trypan blue exclusion assay over time compared to WT cells (16% to 59% cell death), while over 80% of RNase L KO and ABCE1 KD/RNase L KO cells remained viable (Figure 3B). ABCE1 KD clone 2 showed 68% cell death at 24 h after 2-5A transfection (Figure S1D). We then assessed the effects of 2-5A on cell survival in real time of WT, ABCE1 KD, RNase L KO, or ABCE1 KD/RNase L KO cells using dual dyes and an IncuCyte real-time monitoring system for 30 h (described in Methods). The quantitation of cell survival showed that only 10% of ABCE1 KD cells survived after 30 h compared to 47% of WT cells, while RNase L KO and ABCE1 KD/RNase L KO cells were mostly unaffected (Figure 3C,D). These results show that activation of RNase L by 2-5A in cells lacking ABCE1, which increases RNase L activity, also enhances apoptosis compared to WT cells.

### 3.4. ABCE1 Knockdown Augments RNase L-Induced Apoptosis by Enhancing Caspase-3 Activity and Beclin-1 Proteolytic Cleavage

**Figure 4:**
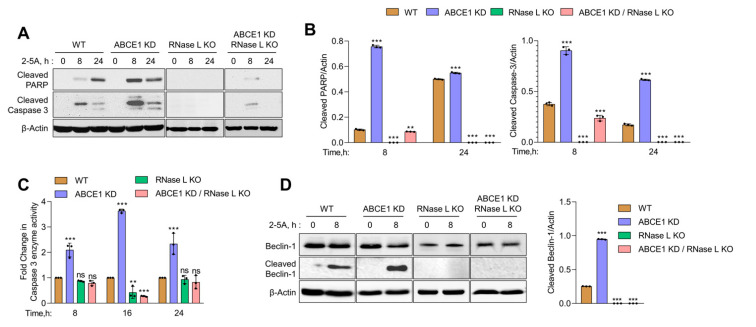
Increased caspase-3 activity and the proteolytic cleavage of Beclin-1 in ABCE1 knockdown cells on RNase L activation by 2-5A. (**A**) Immunoblot analysis of HT1080 WT, ABCE1 KD, RNase L KO, and ABCE1 KD/RNase L KO cells transfected with 10 µM of 2-5A for indicated times and probed with cleaved PARP and cleaved caspase-3 antibodies and normalized to β-actin levels. (**B**) The band intensity was calculated using Image J software and the ratio of cleaved PARP/β-actin or cleaved caspase-3/β-actin was determined and the levels were compared to WT cells. (**C**) Caspase-3/7 enzyme activity was measured in HT1080 WT, ABCE1 KD, RNase L KO, and ABCE1 KD/RNase L KO cells transfected with 10 µM of 2-5A for indicated times and the cleavage of fluorescent caspase-3 substrate was determined. Results shown represent mean ± SD for the experiment performed in triplicate and representative of three independent experiments. (**D**) Immunoblot analysis of HT1080 WT, ABCE1 KD, RNase L KO, and ABCE1 KD/RNase L KO cells transfected with 10 µM of 2-5A for indicated times and probed with Beclin-1 antibodies and normalized to β-actin levels. The band intensity was calculated using Image J software and the ratio of cleaved Beclin-1/β-actin was determined and the levels were compared to WT cells. Results are representative of three independent experiments. WT: wild-type; ** *p* < 0.01; *** *p* < 0.001; ns: not significant.

In previous studies, we showed that small dsRNAs generated by RNase L enzyme activity promote a switch from autophagy to apoptosis by the caspase-mediated cleavage of key autophagy protein, Beclin-1 [13]. Here, we show increased RNase L activity accompanied by early onset of autophagy correlating with a switch to apoptotic cell death in cells lacking ABCE1. Together, these events suggest the involvement of caspase cascade and Beclin-1 cleavage in events leading to enhanced cell death in ABCE1 KD cells. To investigate the activation of caspase-3, we monitored the cleavage of caspase-3 and PARP, both hallmarks of apoptosis, in WT, ABCE1 KD, RNase L KO, and ABCE1 KD/RNase L KO cells after 2-5A transfection at indicated times on immunoblots. The appearance of cleaved PARP was evident by 8 h in ABCE1 KD cells (9-fold more than WT) and remained sustained at 24 h compared to WT cells. As expected, PARP cleavage was barely detectable in RNase L KO and ABCE1 KD/RNase L KO cells. Caspase-3 activity, measured by levels of cleaved caspase-3 on immunoblots, showed a similar temporal profile in ABCE1 KD cells (2.5-fold more than WT at 8 h and 3.6-fold more at 24 h) compared to WT cells and low to no cleavage in RNase L KO cells and ABCE1 KD/RNase L KO cells (Figure 4A,B). We measured caspase-3 enzyme activity in cell lysates using a fluorescent substrate following RNase L activation at indicated times and observed a 2-fold to 3.6-fold increase in ABCE1 KD cells compared to WT cells. No significant caspase-3 enzyme activity was detected in the lysates of RNase L KO cells and ABCE1 KD/RNase L KO cells (Figure 4C). Based on our previous results, we hypothesized that increased apoptosis due to RNase L activation in ABCE1 KD cells could be attributed to caspase-3-mediated cleavage of Beclin-1. Subsequently, using immunoblot analysis, we determined increased cleavage of Beclin-1 (3.8-fold more than WT) 8 h after RNase L activation in ABCE1 KD cells compared to WT cells, which overlapped with increased caspase-3 activity (Figure 4D). No significant modulation of caspase-3 activity or Beclin-1 cleavage was observed in RNase L KO cells and ABCE1 KD/RNase L KO cells. These results suggest that compared to WT cells, increased RNase L activity in ABCE1 KD cells enhances apoptosis by the increased caspase-3-mediated cleavage of Beclin-1 and cells lacking both ABCE1 and RNase L are resistant to apoptosis by 2-5A treatment.

### 3.5. ABCE1 Regulates Autophagy during EMCV Infection

**Figure 5:**
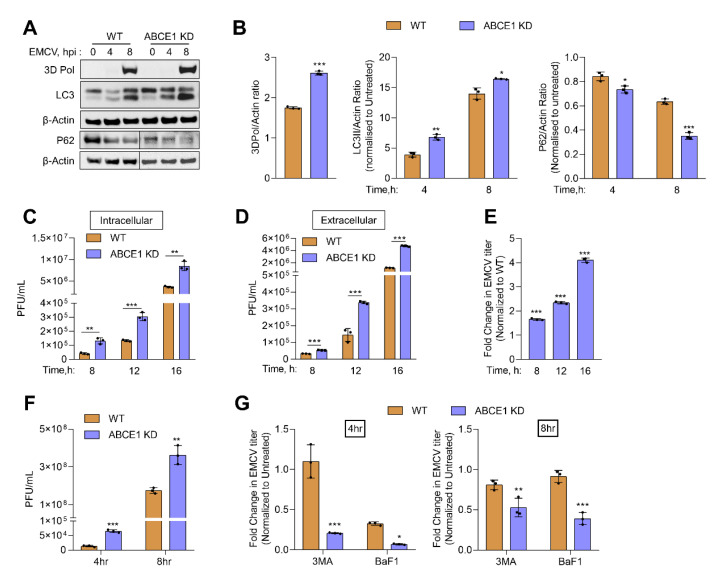
Effect of ABCE1 on autophagy during EMCV infection. HT1080 WT and ABCE1 KD cells were infected with EMCV (MOI of 1.0) and at indicated times (**A**) conversion of unconjugated LC3-I to lipidated LC3-II, degradation of p62, and accumulation of viral protein 3D Pol were monitored on immunoblots and normalized to β-actin levels. (**B**) The band intensity was calculated using Image J software, and the ratio of LC3-II/β-actin, p62/β-actin, or 3D Pol//β-actin was determined and the levels were compared to WT cells. (**C**) Intracellular and (**D**) extracellular titer of EMCV were determined by a plaque assay, and (**E**) the fold change in EMCV titers in supernatants of ABCE1 KD cells was compared to WT cells. Results are representative of three independent experiments. (**F**) EMCV titers in WT and ABCE1 KD cells 4 h or 8 h post infection. (**G**) WT and ABCE1 KD cells were left untreated or were pretreated with 3-MA (5 mM) or bafilomycin A1 (100 nM) 1 h prior to infection with EMCV (MOI of 1.0), and viral titers in the supernatant were determined by a plaque assay. The fold change in the viral yield in WT and ABCE1 KD cells treated with either 3-MA or bafilomycin A1 was compared to untreated samples. Data represent mean ± SD performed in triplicate. WT: wild-type; * *p* < 0.05; ** *p* < 0.01; *** *p* < 0.001.

Previous work has shown that EMCV infection can induce autophagy in an RNase L-dependent manner and at later times of viral growth, inhibiting autophagy reduced EMCV yield [11,12]. Our results show increased autophagy in ABCE1 KD cells, so we determined the impact of ABCE1 on EMCV infection. HT1080 WT and ABCE1 KD cells were infected with EMCV at multiplicity of infection (MOI) of 1. Cells were harvested at 4 h and 8 h post infection, proteins were separated by denaturing polyacrylamide gel electrophoresis, and immunoblots were probed with LC3 and P62 antibodies to monitor autophagy induction. Lysates were also probed with anti-3D-Pol antibody to detect accumulation viral of the viral protein (Figure 5A).

Compared to WT cells, increased conversion of LC3-I to LC3-II was observed in ABCE1 KD cells that correlated with the degradation of the autophagy receptor, P62, as early as 4 h post infection. The increase in LC3-II conversion and P62 degradation continued at 8 h post infection in ABCE1 KD cells (Figure 5B). To test the impact of enhanced autophagy in ABCE1 KD cells on the replication of EMCV, intracellular and extracellular viral titers were determined following the infection of WT and ABCE1 KD cells at indicated times, starting at 4 h post infection (Figure 5C,D). ABCE1 KD resulted in a 1.5-fold to 4-fold increase in levels of both intracellular and released EMCV at all the time points (Figure 5C–E). To determine the effect of autophagy on EMCV replication, WT and ABCE1 KD cells were pretreated with autophagy inhibitors, 3-MA, or bafilomycin A1 prior to EMCV infection. Viral titers were determined 4 h and 8 h post infection from supernatants and compared to untreated cells. ABCE1 KD cells showed higher viral titers compared to WT cells. However, treatment with both autophagy inhibitors reduced EMCV titers more drastically in ABCE1 KD cells at both 4 h and 8 h post infection compared to WT cells (Figure 5F,G). Taken together, these results indicate that ABCE1 plays an important role in regulating EMCV-induced autophagy and that the enhanced autophagy induction in ABCE1 KD cells supports EMCV infection.

## 4. Discussion

The OAS/RNase L antiviral pathway is activated by IFN, produced during viral infections, and the activity of RNase L is regulated by 2-5A synthesized by certain OAS isoforms from cellular ATP. Many RNA and DNA viruses are susceptible to the antiviral effects of this pathway. Accordingly, viruses encode for proteins that antagonize or inhibit one or many steps of this pathway [17,18]. In addition, host cells express proteins that keep the activation of this pathway in check as RNase L cleaves cellular RNAs and inhibits protein synthesis that can be detrimental to cellular homeostasis. This study focused on one of the earliest identified cellular inhibitors of RNase L, ABCE1, also known as RNase L inhibitor (RLI). RLI was characterized as a negative regulator of the OAS/RNase L pathway by antagonizing 2-5A binding and nuclease activity of RNase L [38]. Our results show that the knockdown of ABCE1 in HT1080 fibrosarcoma cells results in increased RNase L activity in cells transfected with its activator, 2-5A, or synthetic dsRNA (poly I:C), which activates OAS to produce 2-5A. Two independent ABCE1 KD clones yielded similar results in the assays we studied, ruling out clone-specific artifacts (Figure S1). A similar increase in RNase L activity in RLI KD cells was observed in prostate cancer cells activated with 2-5A [60]. A decrease in the inhibition of RNase L activity was also observed in Hela cells expressing RLI antisense cDNA [42]. We quantified the differences in RNase L activity across cell lines and samples using the RNA integrity number (RIN) to minimize variability [61]. The effects of ABCE1 on inhibiting RNase L is specific as cells lacking both proteins show no nucleolytic activity. In contrast, another recent study showed the interaction of ABCE1 with Dom34 (Pelota) and RNase L to function as a positive regulator of exogenous RNA decay [40]. It is not clear if transfected exogenous RNAs are processed differently from the endogenous RNAs cleaved in the cellular context and during viral infections. In line with other studies on RNase L activation, in our studies, the ligand 2-5A was delivered by complexing with lipid reagents, in contrast to the electroporation-mediated delivery used by Nogimori et al. [40]. Furthermore, basal levels of OAS proteins and RNase L vary by cell types and are determinants of IFN induction during viral infection, including EMCV [64]. Hela M cells are deficient in endogenous RNase L activity and have been used to reconstitute the expression of RNase L mutants [65,66,67]. It is not clear if the variable levels of endogenous RNase L in cell lines, along with differences in the dose and mode of delivery of 2-5A, can explain the differences in the two studies, and further investigation will be required. ABCE1 is evolutionarily conserved in archaea and eukaryotes, compared to the limited function of RNase L in higher vertebrates, suggesting more fundamental roles. Accordingly, recent studies have shown ABCE1 function in regulating translation, ribosome recycling, and ribosome homeostasis. Depletion of ABCE1 in yeast, *Drosophila,* and mammalian cells have shown a marked reduction in polysomes and accumulation of mRNA-free 80S monomers [51,68]. It is likely that in cells depleted of ABCE1, cellular RNAs inclusive of mRNAs are more accessible to RNase L for cleavage, causing enhanced degradation. Similar to other results, our data support the role of ABCE1 as an inhibitor of RNase L activity.

Activation of RNase L during a viral infection or directly by 2-5A transfection induced autophagy only in cells expressing WT RNase L and not in RNase L KO Mouse embryonic fibroblasts (MEFs) or cells with RNase L knockdown. Reconstituting RNase L KO cells with a RNase L mutant lacking enzyme activity did not restore autophagy, implicating RNA cleavage as a triggering event [11,12]. Our results show that RNase L activation in ABCE1 knockdown cells significantly enhanced autophagy at earlier time points that correlate with RNase L activity compared to parental WT cells or cells lacking RNase L or both proteins. RNase L cleaves single-stranded regions of viral and cellular RNAs, including rRNA, to produce short dsRNAs with signaling roles. RNase L-mediated rRNA cleavage can inhibit protein translation and disassembly and turnover of polysomes similar to observations made in ABCE1-depleted yeast, *Drosophila*, and mammalian cells. Therefore, the activation of RNase L in ABCE1 knockdown cells can synergistically enhance the accumulation of non-translating ribosomes that may be sequestered in autophagosomes by specialized autophagy, ribophagy, and serve as a recycling pathway.

RNase L participates in the cross-talk between autophagy and apoptosis. Depending on the cell type and the expression levels of apoptotic and anti-apoptotic proteins, RNase L activates caspase-3 to induce apoptosis [13,60,63,64]. Our previous studies showed that dsRNAs produced by RNase L induces caspase-3, which in turn cleaves autophagy protein, Beclin-1, to promote a switch from autophagy to apoptosis [13]. Here, we have demonstrated that RNase L activation in ABCE1 knockdown sensitizes cells to apoptosis and shows significantly reduced cell proliferation. Our results show a close correspondence of increased RNase L activity with caspase-3 enzyme activity, as observed previously in prostate cancer cells with ABCE1 knockdown. Here, we have shown that caspase-3 activity and cleavage of PARP is consistent with caspase-3-mediated cleavage of Beclin-1 observed at the same time points after RNase L activation. The knockdown of ABCE1 expression in human esophageal cancer, breast cancer, and small cell lung cancer cells causes reduced proliferation, increased apoptosis, migration, and invasion; however, the role of RNase L inhibition in mediating these effects remains to be determined [53,54,55]. It is likely that mammals may have evolved additional roles of ABCE1 in regulating the tumor suppressor roles of RNase L by regulating RNase L enzyme activity.

ABCE1 has diverse roles during viral infections. The initial characterization of ABCE1 supported an inhibition of antiviral role of IFN during infection with EMCV and HIV. In these studies, ABCE1 was transcriptionally induced by virus infection and not IFN. In both instances, overexpression of ABCE1 inhibited RNase L activity and enhanced virus production; however, decreasing ABCE1 levels correlated with decreased viral yield [41,42]. Based on recent studies, ABCE1 may serve dual roles in HIV pathogenesis. Overexpression causes a decrease in RNase L activity and increased HIV production. In contrast, antisense ABCE1 (RLI) construct expression reverses inhibition of RNase L activity, with a corresponding decrease in HIV titers [41]. Additionally, ABCE1 (HP68) was shown to be required for immature HIV-1 capsid assembly [44]. Recently, ABCE1 has been shown to interact with gag and cellular protein, DDX6, to facilitate HIV-1 immature capsid assembly using RNA granules [43,69,70]. In other studies, ABCE1 was identified in a genome-wide screen as a proviral host protein needed for the efficient translation of measles (MeV), mumps (MuV), and RSV mRNA and not cellular RNAs [45]. Many viruses induce translation shut-off by perturbing mRNA or protein synthesis. However, paramyxovirus relies on ongoing protein synthesis and requires the ribosome recycling function of ABCE1. The polycistronic paramyxovirus mRNA could cause altered termination of ribosome, and hence the rigid requirement of ABCE1 to recycle ribosomes in sustaining viral replication. The initiation of translation during EMCV infection is cap independent due to its internal ribosome entry sites (IRES) that bind eIFs to directly recruit the 40S ribosomal subunit [71]. The precise role of ABCE1 in the translation of EMCV proteins will need further investigation. However, we observe an increase in the accumulation of EMCV proteins in ABCE1 KD cells detected on immunoblots by 3D polymerase antibodies (Figure 5A). Previous studies have shown that autophagy induced by RNase L during SeV, EMCV, or VSV infections is antiviral in the early stages of infection and at later time points, subversion of autophagy promotes viral growth [11,12]. In our studies, early onset of autophagy in ABCE1 knockdown cells during EMCV infection increased virus yield. These observations support studies that indicate that autophagy promotes EMCV replication and the RNA replication during infectious cycle occurs on autophagosome-like membranes. The effect of autophagy on EMCV replication varies by viral strain, cell type, and multiplicity of infection (MOI) and length of infection. At lower MOI and single replication cycle, autophagy induced in WT cells has antiviral roles and at later times promotes viral growth [11,12]. In ABCE1 knockdown cells, early onset of autophagy facilitates replication, at levels higher than WT cells, and a switch to apoptosis possibly contributes to viral dissemination and perpetuation. While not explored here, additional roles of ABCE1 in ribosome recycling, regulating translation, and ribosome homeostasis may together contribute to EMCV pathogenesis. Our studies identify ABCE1 as a regulator of the OAS/RNase L pathway by balancing and fine-tuning the RNase L activity that can impact the outcomes of viral infections.

## Data Availability

The data presented in this study are available on request from the corresponding author.

## Supplementary Materials

The following are available online at https://www.mdpi.com/1999-4915/13/2/315/s1, Figure S1: Characterization of ABCE1 knockdown clone 2.
