# Supplementary material for: ABCE1 Regulates RNase L-Induced Autophagy during Viral Infections
Source: Viruses. 2021 Feb 18;13(2):315. doi: 10.3390/v13020315 (PMC7922175; doi:10.3390/v13020315)
Supplement: Supplementary file 1 [file viruses-13-00315-s001.zip › viruses-1102581-supplementary.pdf]

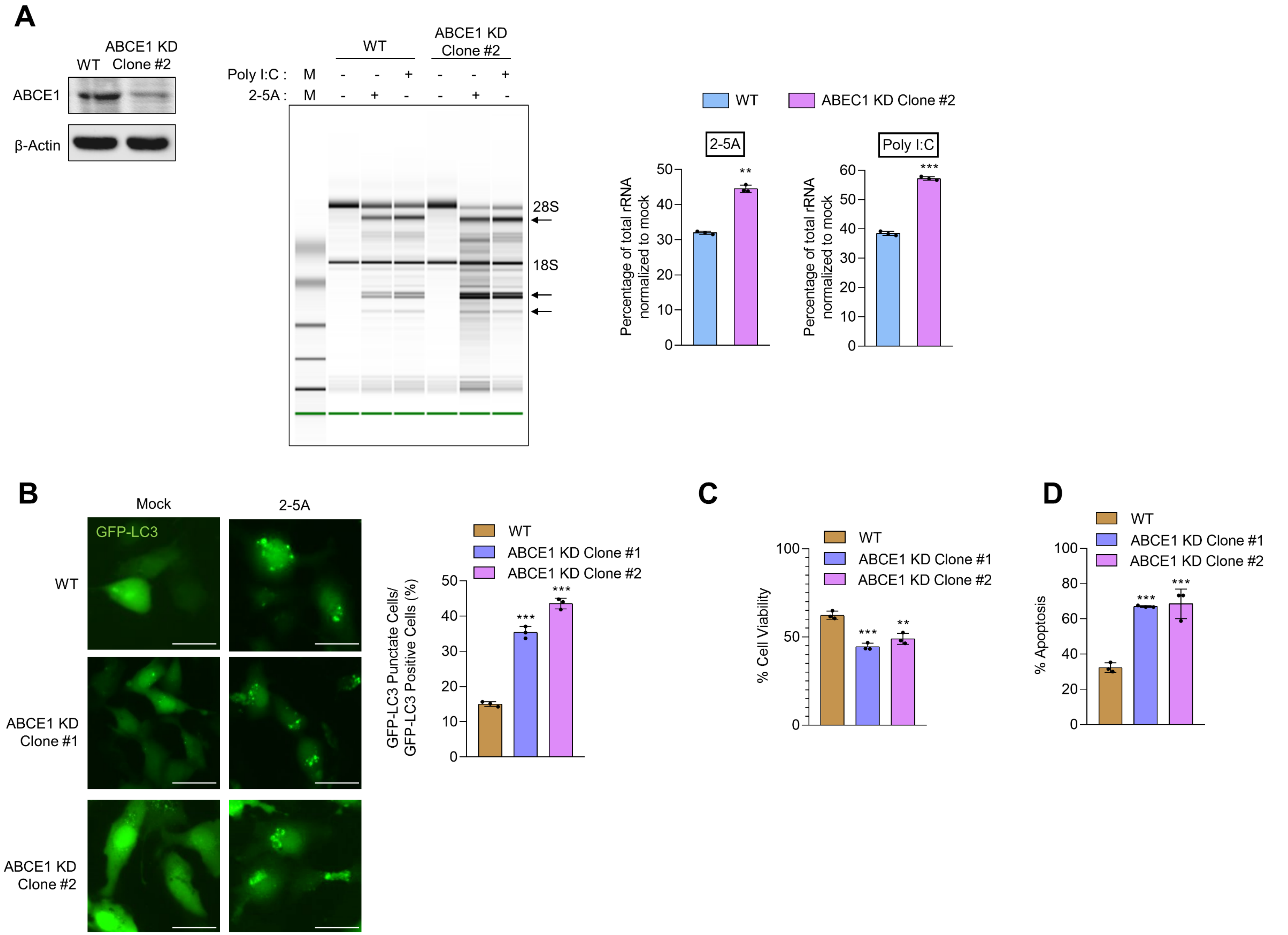

Figure S1. (**A**) Knockdown of ABCE1 levels in clone 2 using shRNA in HT1080 WT cells was verified in cell lysates by immunoblotting using specific antibodies. WT and ABCE1 KD clone 2 cells were transfected with 2–5A (10 μM) or 2 μg/mL of Poly I:C for 6 h and RNase L-mediated cleavage of rRNA (arrows) was analyzed on RNA chips using the Agilent Bioanalyzer 2100. Cleavage of rRNA was quantitated and normalized to mock treated samples. (**B**) HT1080 WT and ABCE1 KD clone 1 and clone 2 cells expressing GFP-LC3 were mock transfected or transfected with 2–5A (10 μM) and the formation of GFP-LC3 puncta was microscopically imaged and the percentage of GFP+ cells showing puncta formation compared with mock-treated cells was analyzed. Scale bar 50 μm. HT1080 WT and ABCE1 KD clone 1 and clone 2 cells were transfected with 10μM of 2–5A for indicated times and (**C**) Cell Viability was measured using MTT reagent, (**D**) Percent apoptotic cells was determined by Trypan blue dye exclusion assay. Experiments performed in triplicate and shown as mean ± SD and compared to WT cells. WT: wild-type; **, $p < 0.01$; ***, $p < 0.001$.
